# Mitochondrial permeability transition in protozoan parasites: what we learned from *Trypanosoma cruzi*

**DOI:** 10.1038/cddis.2017.431

**Published:** 2017-09-21

**Authors:** P L Bustos, A E Perrone, N A Milduberger, J Bua

**Affiliations:** 1 Instituto Nacional de Parasitología ‘‘Dr. Mario Fatala Chabén’’- A.N.L.I.S. Malbrán, 568 Paseo Colon Avenue, C1063AC S, Buenos Aires, Argentina; 2 Consejo Nacional de Investigaciones Científicas y Técnicas, Buenos Aires, Argentina; 3 CAECIHS, Universidad Abierta Interamericana, Av. Montes de Oca 745, 2º piso, Buenos Aires C1270AAH, Argentina

Regulated cell death (RCD) involves a genetically encoded molecular machinery, which can be altered by means of pharmacologic and/or genetics interventions targeting the key components of such machinery. RCD often occurs in a delayed manner and is initiated in the context of adaptive responses that unsuccessfully attempt to restore cellular homeostasis. It is important to mention that the term RCD includes both physiological instances of death, referred to as ‘programmed cell death’, but also death processes that occur in pathological contexts. Our comprehension of cell death subroutines has progressed significantly, as the main molecular events underlying these mechanisms have been elucidated.^1^

A variant of RCD that often manifests with necrotic morphotype critically relies on Cyclophilin D (CyPD), a mitochondrial matrix peptidyl-prolyl isomerase, which is encoded by the *Ppif* gene. At present, CyPD is the unique genetically confirmed component of the permeability transition pore complex (PTPC) in the mammalian system,^2, 3^ a supramolecular complex operating at the junctions between the inner and outer mitochondrial membranes that may cause the ‘mitochondrial permeability transition’ (MPT), an abrupt increase in the permeability of the inner membrane to small solutes, triggered by cytosolic Ca^2+^ overload and/or oxidative stress.^4^ The importance of CyPD for MPT has been recognized for a long time in mammalian systems, mostly due to the consistent cytoprotective effects mediated *in vitro* and *in vivo* by Cyclosporin A (CsA), an immunosuppresive undecapeptide that acts as a CyP inhibitor.^4^ Moreover, both the administration of CsA and the genetic ablation of the *Ppif* gene in a knockout mice model (known as Ppif^−/−^ mice) have been shown to limit necrotic cell death, *in vitro* as well as *in vivo*, in a variety of pathophysiological settings, including ischemia−reperfusion injuries of the heart, brain and kidney (reviewed in Galluzzi *et al*^1^). Once MPT has been established, it seals the cell fate independently of caspase activation. ‘MPT-driven RCD’ should be used for cell death instances whose course can be influenced by the genetic or pharmacological inhibition of CyPD. Perhaps, CyPD's central role in MPT-driven RCD reflects its ability to control the Ca^2+^ buffering capacity of the mitochondrial network, although this hypothesis has not been yet fully addressed.^1^

A lot of research has been done in mammals, but still very little is known for protozoan parasites, one of the most ancient phylogenic branches of unicellular eukaryotes. Although the benefits of RCD in unicellular organisms are less evident than in mammalian tissues, there are increasing numbers of reports that describe that some unicellular organisms undergo RCD under certain conditions. A more precise description of unicellular death would be informative in the comprehension of how cell death has evolved in higher eukaryotes.

Moreover, as Cyclosporin A and its non-immunosuppresive analogs are known to exhibit anti-parasitic effects on a wide range of organisms, including several protozoan parasites of medical importance, a profound knowledge of their cyclophilin repertoire and the possibility of the MPT-driven RCD pathway present in these organisms represents a challenging field to be explored.

The response of the RCD phenotype to various stimuli has been measured in protozoan parasites. In Table 1, some features observed in kinetoplastids are listed. A vast number of stimuli have been used to challenge these organisms, with different outcomes. The most common cell death features found in mammalian tissues were seen to occur, such as phosphatidylserine exposure, DNA degradation and mitochondrial membrane depolarization. However, Cyclosporin A has been reported to have effect as an RCD inhibitor only against *Trypanosoma cruzi*, where we observed that parasites grown in an oxidative stress environment with H_2_O_2_ underwent cell death, showing typical features such as DNA degradation, ROS production, cytochrome *c* release into the cytosol after induction and sensitivity to CsA inhibition, suggesting that a *T. cruzi* mitochondrial cyclophilin could be present in a MPT-like structure in this protozoan parasite.^5^

*T. cruzi* is a unicellular protozoan parasite that infects 7–8 million people in South America as well as in other parts of the world through migrations from endemic areas.^6^ The *T. cruzi* infection can evolve into Chagas disease, a potential life-threatening illness.^7^ Our research group has described the *T. cruzi* CyP gene family and reported the expression of several parasite cyclophilins that exhibited enzymatic PPIase activity, inhibited by CsA.^8^

In a report published in *Cell Death Discovery*, we identified that a homolog of mammalian CyPD is expressed in *T. cruzi*, named *Tc*CyP22. This protein was localized to the parasite mitochondrion in the three stages of the parasite life cycle, as expected. Interestingly, in parasites overexpressing *Tc*CyP22, an increased susceptibility to hydrogen peroxide effects was observed, demonstrating that this protein is directly involved in parasite RCD.^9^

To our knowledge, this was the first identification of a homolog of a CyPD in a protozoan parasite and shows that the MPT-driven RCD could be an evolutionarily well-conserved pathway from this ancient eukaryote. However, whether CyPD homologs are also present in the other protozoan parasites remains to be elucidated.

The study of parasitic protozoa during infections in the insect and mammalian hosts could provide useful information about natural cell death. These insights could ultimately lead to the identification of key regulatory or executioner molecules that are central to RCD. Such discoveries would potentially provide the basis of novel therapeutic strategies. Further study of protozoan parasites' death process will be of significance in a greater understanding of the interaction between the parasite and its host, and also cell death mechanism in general.

## Figures and Tables

**Table 1:** Cell death features described for kinetoplastid parasites, including the use of Cyclosporin A (CsA) as an RCD inhibitor

	***Trypanosoma cruzi***	***Trypanosoma brucei***	***Leishmania*spp**
*RCD induction*	Yes	Yes	Yes
Stimulus	H_2_O_2_^5, 10^/fresh human serum^10^/starvation^11^	Prostaglandins^12^	H_2_O_2_^13^
			
*Morphological and biochemical features*	Yes	Yes	Yes
Phosphatidylserine exposure	Yes^5, 10, 11^	Yes^12^	nf
Cytochrome c release	Yes^5, 10^	nf	Yes^13^
Mitochondrial membrane potential loss	Yes^5, 10^	Yes^12^	nf
Nucleic acid cleavage assays	Yes^5, 10^	Yes^12^	Yes^13^
Cyclosporin A inhibition	Yes^5, 9^	nf	Yes^14^
Mitochondrial permeability transition pore structure suggested	Yes^5, 9^	nf	Yes^14^
Cyclophilin gene family description	Yes^8^	nf	Yes^15^
Cyclophilin D homologue	Yes^9^	nf	nf

Abbreviation: nf, not found
